# Pilot drive-test LTE handover dataset for radio measurement analysis in urban Bangladesh

**DOI:** 10.1016/j.dib.2026.113220

**Published:** 2026-09-03

**Authors:** M.M. Sadman Shafi, K.M. Istiaque, Shafayet Sadik Sowad, Tasnia Siddiqua Ahona, Mubtasim Labib, Yasin Hasan Saad, Md Nazakat Eram, Mohammad T. Kawser

**Affiliations:** Department of Electrical and Electronic Engineering, Islamic University of Technology, Board Bazar, Gazipur, 1704, Bangladesh

**Keywords:** Mobility management, Handover optimization, Radio access network, Signal quality, LTE measurement data, Network performance evaluation, Real-world dataset, Wireless communication

## Abstract

This article presents a pilot Long Term Evolution (LTE) drive-test dataset collected from a real urban cellular network in Dhaka, Bangladesh. The dataset contains field measurements of radio signal conditions, Layer-3 measurement reports, and handover statistics obtained using professional drive-testing tools including XCAL-M software and Samsung Exynos-based user equipment. Measurements were recorded during multiple drive-test sessions along a 13 km urban route, producing timestamped observations of key radio parameters such as Reference Signal Received Power (RSRP), Reference Signal Received Quality (RSRQ), Carrier-to-Interference-plus-Noise Ratio (CINR), and serving/neighbor cell identifiers. The dataset is organized into raw and processed directories containing dynamic radio management (DRM) log files, Layer-3 measurement reports, handover event statistics, and tabular datasets to support reproducibility and flexible analysis. The processed dataset demonstrates an example preprocessing workflow including filtering, timestamp alignment, interpolation of missing signal values, and handover labeling with Time-to-Trigger (TTT) context. Due to the limited size of the dataset, it is primarily intended for exploratory analysis, mobility characterization, small-scale machine learning experiments, reinforcement learning environments, and educational research in cellular network engineering. By providing both raw drive-test logs and structured datasets, this resource supports reproducible studies of LTE handover behavior and urban radio environments.

Specifications Table

**Table:** 

Subject	Electrical Engineering
Specific subject area	Telecommunication, LTE Networks, Wireless Communication, Cellular Communication
Type of data	.csv (raw and processed datasets), .txt (measurement reports, event statistics), .drm (XCAL-M binary log files)
Data collection	Data were collected via structured drive tests conducted in Dhaka, Bangladesh using XCAL-M (Accuver) drive test software, Samsung Galaxy S10 (Exynos), and an Arduino Uno with Neo-7 M GPS module. Measurements were recorded during peak evening hours across a 13 km urban route and include Layer 3 signaling events, signal strengths, User Equipment (UE) velocity, and handover logs. Preprocessing included cleaning, filtering, timestamp alignment, and handover labeling with a TTT window. The processed dataset illustrates an example workflow for exploratory data analysis and small-scale machine learning experiments, while the original raw logs remain available for independent processing by users.
Data source location	Country: Bangladesh City: Dhaka Route: Le Meridien (Uttara) to BRAC University campus
Data accessibility	Repository name: Mendeley Data Data identification number: 10.17632/n2pvmtyn2j.2 Direct URL to data: https://data.mendeley.com/datasets/n2pvmtyn2j/2 All raw data underlying the tables, figures, graphs, and charts presented in this article have been deposited in the publicly accessible Mendeley Data repository (DOI: 10.17632/n2pvmtyn2j.2). The repository contains the original XCAL-M DRM diagnostic logs, Layer-3 measurement reports, handover event statistics, raw CSV measurement files, and processed datasets in open, non-proprietary CSV and TXT formats. All visualizations reported in this manuscript are directly reproducible from these deposited data. The processed datasets are derived from the released raw measurements using the preprocessing workflow described in Section 4.6, and no additional raw data are withheld behind institutional access restrictions or paywalls.
Related research article	None

## 1 Value of the Data

• This dataset is one of the first publicly available LTE mobility datasets collected from real network environments in Bangladesh, where such granular handover-related data has not been documented or shared before. Its novelty stems from the combination of a geographically underrepresented region, fine-grained handover event logging, and the inclusion of UE velocity information—features rarely found together in existing LTE mobility datasets and urban radio environment studies [1]. In addition to addressing a regional data gap, this dataset can support comparative and benchmarking studies with similar LTE mobility datasets from Europe and North America, contributing to a global understanding of cellular performance trends in evolving wireless ecosystems [2]. This addresses the limited availability of publicly accessible LTE mobility datasets from South Asia and provides a geographically representative benchmark for researchers investigating mobility management in underrepresented urban cellular environments.
• The dataset includes timestamped serving-cell and neighbor-cell radio measurements (RSRP, RSRQ, CINR) together with handover event statistics collected using professional drive-test software. These measurements allow researchers to examine real-world LTE radio conditions and handover behavior in dense urban deployments. The dataset is particularly useful for wireless communication researchers, radio access network (RAN) engineers, and mobility-management researchers seeking to analyze real-world LTE handover behavior and radio propagation characteristics.
• Both raw drive-test logs and processed tabular datasets are provided. The raw logs enable researchers to independently reconstruct the dataset or apply alternative preprocessing strategies, while the processed datasets demonstrate a reproducible example workflow for signal analysis and handover labeling. By publishing both the raw diagnostic logs and structured datasets, the work promotes transparency and reproducibility in cellular network measurement research. The availability of both raw and processed data also enables independent preprocessing, feature extraction, protocol-level validation, and reproducibility studies using alternative analytical pipelines.
• The dataset supports multiple reuse scenarios, including benchmarking handover prediction models, validating reinforcement learning and contextual bandit algorithms, comparative evaluation of mobility-management methods, statistical characterization of LTE radio environments, and small-scale machine learning studies. It may also serve as an educational resource for coursework related to cellular network measurements and mobility management. The dataset has also been used in studies on reinforcement-learning-based handover optimization, deep reinforcement learning, contextual bandit learning, and anomaly-based handover prediction, demonstrating its applicability to a range of LTE mobility analysis tasks [[3], [4], [5], [6]].
• The dataset was collected from a single LTE network operator along one urban drive-test route in Dhaka, Bangladesh, during three measurement sessions. Accordingly, it is intended as a transparent pilot dataset for reproducible LTE mobility and handover studies rather than a comprehensive representation of nationwide or multi-operator network conditions. Future users should interpret the dataset within this scope when conducting comparative analyses or developing mobility-management algorithms.

## 2 Background

Publicly available cellular drive-test datasets remain relatively limited, particularly for developing regions such as South Asia. Most studies investigating LTE mobility and handover performance rely on either simulation environments or proprietary operator measurements that are not publicly accessible. This limits reproducibility and comparative evaluation of mobility-management algorithms.

Several prior studies have reported LTE or cellular measurement datasets, although many focus on different objectives or omit detailed handover-related information. A study from Nigeria’s Sabon Gari Market provided LTE performance insights but lacked structured handover event representation [7]. An Austrian highway dataset focused primarily on throughput and data-rate analysis without including raw RRC measurements or detailed mobility parameters [8]. A Nigerian LTE assessment reported network quality indicators but lacked timestamped granularity and reusable structured datasets [9]. A 2 G cellular dataset captured RF parameters but did not include LTE-specific mobility metrics [10]. Another 4 G trace dataset provided throughput, channel, and contextual KPIs across mobility scenarios, but it did not specifically emphasize handover behavior or structured serving–neighbor cell relationships [11]. Similarly, a publicly released LTE trace dataset containing short-duration measurements sampled at 100 ms focused mainly on channel quality analysis and data-cleaning workflows rather than handover characterization [12]. A vehicular mobile bandwidth dataset collected in Australia included download-rate traces with GPS and operator information but did not provide radio-layer LTE measurements such as RSRP, RSRQ, or handover event statistics required for radio mobility analysis [13].

In contrast, the dataset introduced in this work combines LTE drive-test measurements, Layer-3 signaling information, serving and neighbor cell parameters, and structured handover event records collected from a real urban cellular network environment in Dhaka, Bangladesh [14]. The processed datasets additionally include timestamp-aligned records and TTT-aware handover annotations derived from the original measurement logs [15]. Because the dataset contains a relatively small number of observations compared with large-scale operator datasets, it is not intended for training large deep learning models. Instead, it represents a pilot drive-test measurement dataset collected from a real LTE network environment in Bangladesh. Its primary objective is to document real-world radio measurements and handover behavior observed during controlled drive-test sessions.

Several prior studies have reported LTE or cellular measurement datasets, although many focus on different objectives or omit detailed handover-related information. A comparative summary of representative publicly available LTE datasets is provided in Table 1.

**Table 1:** Comparison of representative publicly available cellular mobility dataset.

Reference	Technology	Approx. Dataset Size	Radio Measurements	GPS / Mobility	Protocol-Layer Information	Handover Annotations	Raw Diagnostic Logs	Geographic Coverage
LTE User Equipment Measurements [7]	LTE	∼47,000 measurements	RSRP, RSRQ, RSSI, SNR, CQI, Throughput, Cell IDs	✓	✗	✗	✗	Nigeria
Austrian Highway Dataset [8]	LTE	Not reported	Throughput, data rate	✓	✗	✗	✗	Austria
Nigerian LTE Assessment [9]	LTE	Not reported	Network quality indicators	Partial	✗	✗	✗	Nigeria
Open Mobile Communications Drive Test Dataset [10]	2G/3G/ 4 G (LTE)	>267,000 drive-test samples	Radio measurement, throughput, cell information	✓	✗	✗	✗	Multi-country
Raca et al. [11]	4 G (LTE)	∼835,000 measurements	CQI, throughput, contextual KPIs	✓	✗	✗	✗	Europe
Meixner et al. [12]	LTE	377 traces (>1.1 million signal samples)	RSRP, RSRQ	✓	✗	✗	✗	Austria, Germany, Italy
Vehicular LTE Dataset [13]	3G/4 G (LTE)	56,754 measurements	Bandwidth measurements	✓	✗	✗	✗	Australia
Proposed Dataset	LTE	2155 processed records (raw logs included)	Serving/Neighbour Cell RSRP, RSRQ, CINR, Cell IDs	✓ (Datasets 2 & 3)	✓ (Layer-3 Measurement Reports)	✓	✓ (XCAL-M DRM logs)	Bangladesh

***Note:*** The proposed dataset also includes Event Statistics files, Measurement Reports, and raw XCAL-M DRM logs, enabling independent reconstruction of the processed datasets. Dataset 1 contains radio measurements only, whereas Datasets 2 and 3 additionally include GPS-based mobility information.

## 3 Data Description

### 3.1 Dataset overview

The dataset is structured into multiple directories to support different stages of analysis, from raw signal capture to machine learning-ready formats. Each folder serves a distinct purpose—ranging from unprocessed CSV logs to preprocessed datasets, Layer 3 signaling messages, and original binary logs. This modular organization ensures transparency, reproducibility, and ease of access for researchers seeking to validate or extend LTE handover studies. An overview of the dataset folder structure is provided in Table 2.

**Table 2:** Overview of the repository structure showing the five data folders and their brief descriptions.

Folder	Description
**Parent Dataset**	Raw CSVs from drive tests (3 sets)
**Processed Dataset**	Cleaned, interpolated, ML-ready CSVs
**Measurement Reports**	Layer 3 logs (per file)
**Event Statistics**	Handover attempts, outcomes, timestamps
**DRM Files**	Original .drm logs for full reconstruction

The repository contains five complementary data components that represent successive stages of the same measurement campaign. The DRM files (.drm) preserve the original XCAL-M diagnostic traces, from which Measurement Reports (.txt) and Event Statistics (.txt) were exported. The Parent Datasets (.csv) contain the raw tabular radio measurements extracted from these logs, while the Processed Datasets (.csv) provide timestamp-aligned, cleaned and handover-labeled versions derived from the parent datasets. This organization enables users to reproduce the complete workflow from raw diagnostic logs to analysis-ready tables.

The raw datasets consist of Dataset_1 with 7 files containing 11 columns and a total of 1480 data points, Dataset_2 with 2 files containing 14 columns and 1521 data points, and Dataset_3 with 3 files also containing 14 columns and 1072 data points. After processing, Dataset_1 was reduced to 922 data points while retaining 11 columns, Dataset_2 to 592 data points with 14 columns, and Dataset_3 to 641 data points with 14 columns.

The three datasets correspond to separate drive-test sessions conducted on different days. Due to temporary hardware constraints during the first measurement session, Dataset_1 contains radio measurement parameters and handover information but does not include GPS-based mobility parameters such as latitude, longitude, or speed. Consequently, Dataset_1 primarily represents a radio measurement dataset for signal analysis and handover statistics.

Datasets 2 and 3 include additional mobility-related variables (UE speed and geographic coordinates) collected using a GPS module, allowing limited mobility-aware analysis. This variation between datasets reflects practical constraints during field measurements but also enables comparative studies between purely radio-based measurements and datasets that include mobility information.

The processed datasets were generated from the parent CSV files through timestamp alignment, cell-aware interpolation of short missing signal intervals, and handover label generation using the Time-to-Trigger (TTT) procedure; the complete preprocessing methodology is described in Section 4.6.

To provide a clearer understanding of the dataset's structure and features, we present both the raw and processed views of the collected data. The column names and target variable used across the datasets are summarized in Table 3. The raw data files, collected over three drive test sessions, are described in Table 4, which shows the number of columns and total data points across multiple CSV files. The processed dataset, which has been cleaned, filtered, and interpolated for machine learning applications, is presented in Table 5. Each processed dataset is available in two versions: version 1 includes preprocessing with a 320 ms time-to-trigger constraint, while version 2 is generated without applying this constraint. The number of handover events observed in parent dataset, total distance traveled, and average distance per handover instance for each day of testing are outlined in Table 6.

**Table 3:** Dataset columns.

Features	Timestamp
	Longitude
	Latitude
	Speed (km/h)
	Serving Cell ID
	Serving Cell RSRP
	Serving Cell RSRQ
	Serving Cell CINR-1
	Serving Cell CINR-2
	Serving Cell CINR
	Neighbour Cell ID
	Neighbour Cell RSRP
	Neighbour Cell RSRQ
Target Variable	Handover Trigger

**Table 4:** Raw parent datasets extracted from three drive-test sessions.

Dataset Folder	Dataset Names	Number of Columns	Data Points
Dataset_1	File 1	11	132
	File 2	11	320
	File 3	11	74
	File 4	11	46
	File 5	11	243
	File 6	11	11
	File 7	11	654
Dataset_2	File 1	14	723
	File 2	14	798
Dataset_3	File 1	14	160
	File 2	14	339
	File 3	14	573

**Table 5:** Processed datasets derived from the parent CSV files.

Dataset Names	Number of Columns	Data Points
Dataset_1_ver_1	11	922
Dataset_1_ver_2	11	922
Dataset_2_ver_1	14	592
Dataset_2_ver_2	14	592
Dataset_3_ver_1	14	641
Dataset_3_ver_2	14	641

**Table 6:** Handover Instances with respect to travelled distance.

Time Period	Handover Count	Total Distance Travelled (km)	Distance Travelled per Handover in meter
Day 1	129	26.21	203
Day 2	95	25.54	268
Day 3	107	27.28	254

The dataset contains three Carrier-to-Interference-plus-Noise Ratio (CINR) fields: Serving Cell CINR-1, Serving Cell CINR-2, and Serving Cell CINR. Serving Cell CINR-1 and Serving Cell CINR-2 represent the lower and upper CINR measurements reported by the XCAL-M diagnostic logs for the serving cell, while Serving Cell CINR is the arithmetic mean of these two values, providing a single representative CINR metric for subsequent analysis. Including both individual measurements together with their averaged value preserves the original diagnostic information while also providing a consolidated signal quality metric for users who require a single CINR indicator.

Descriptive statistics for each dataset are provided in Tables 7, 8, and 9, which correspond to Dataset_1, Dataset_2, and Dataset_3, respectively. These statistics include signal strength parameters such as RSRP, RSRQ, and CINR for both serving and neighboring cells, as well as UE speed where applicable. This layered presentation ensures transparency in both the raw collection process and the structured post-processing steps.

**Table 7:** Descriptive statistics of Dataset_1.

Statistic	Serving Cell RSRP	Serving Cell RSRQ	Serving Cell CINR	NeighCell RSRP	NeighCell RSRQ
Count	922	922	922	922	922
Mean	47.04	12.41	−2.02	45.65	9.25
Std	13.04	7.23	10.81	11.62	7.74
Min	13	0	−30	1	0
Max	78	51	31.5	77	45

**Table 8:** Descriptive statistics of Dataset_2.

Statistic	Speed (km/h)	Serving Cell RSRP	Serving Cell RSRQ	Serving Cell CINR	NeighCell RSRP	NeighCell RSRQ
Count	592	592	592	592	592	592
Mean	3.33	52.13	11.19	−1.35	50.30	7.79
Std	3.98	9.31	6.05	5.86	9.12	6.03
Min	0	21	0	−15	22	0
Max	14.49	76	24	25	76	24

**Table 9:** Descriptive statistics of Dataset_3.

Statistic	Speed (km/h)	Serving Cell RSRP	Serving Cell RSRQ	Serving Cell CINR	NeighCell RSRP	NeighCell RSRQ
Count	641	641	641	641	641	641
Mean	23.45	52.19	11.82	−1.06	51.05	7.69
Std	14.61	10.67	7.06	8.31	10.08	6.28
Min	0	19	0	−30	18	0
Max	63	78	31	25	80	46

### 3.2 Quantitative analysis of dataset

**Fig. 1:**
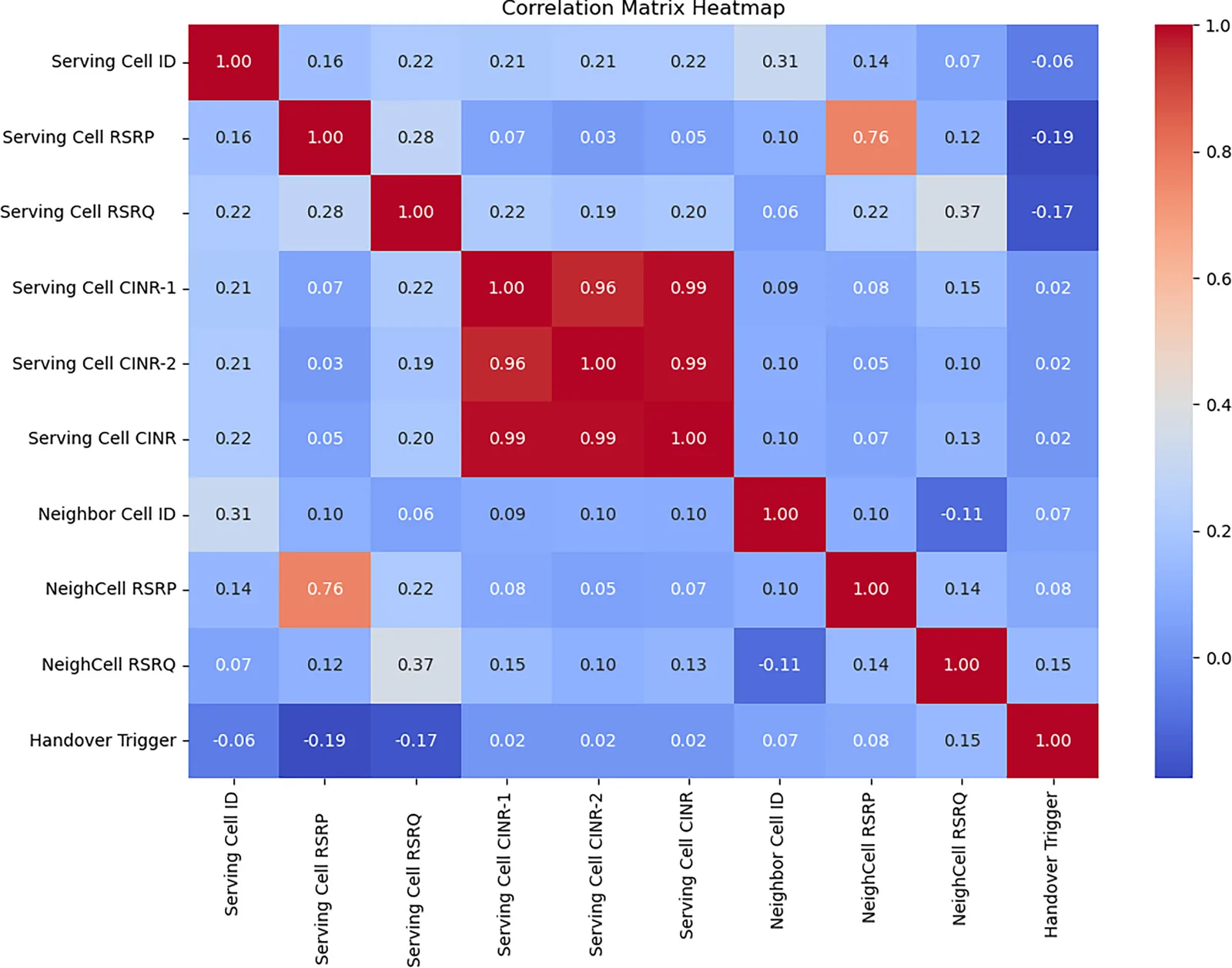
Correlation matrix illustrating the relationships among the network parameters in Dataset_1.

**Fig. 2:**
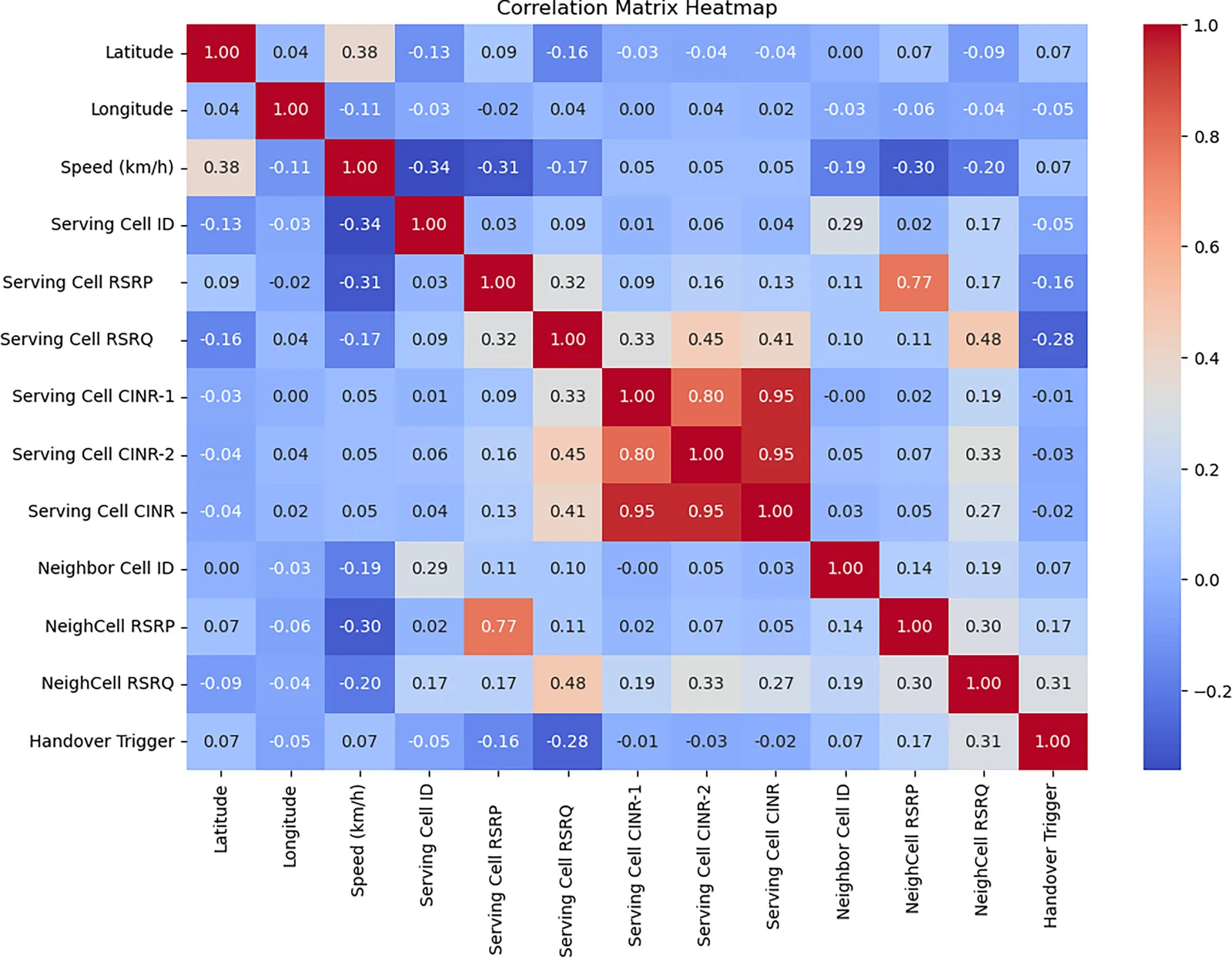
Correlation matrix illustrating the relationships among the network parameters and mobility variables in Dataset_2.

**Fig. 3:**
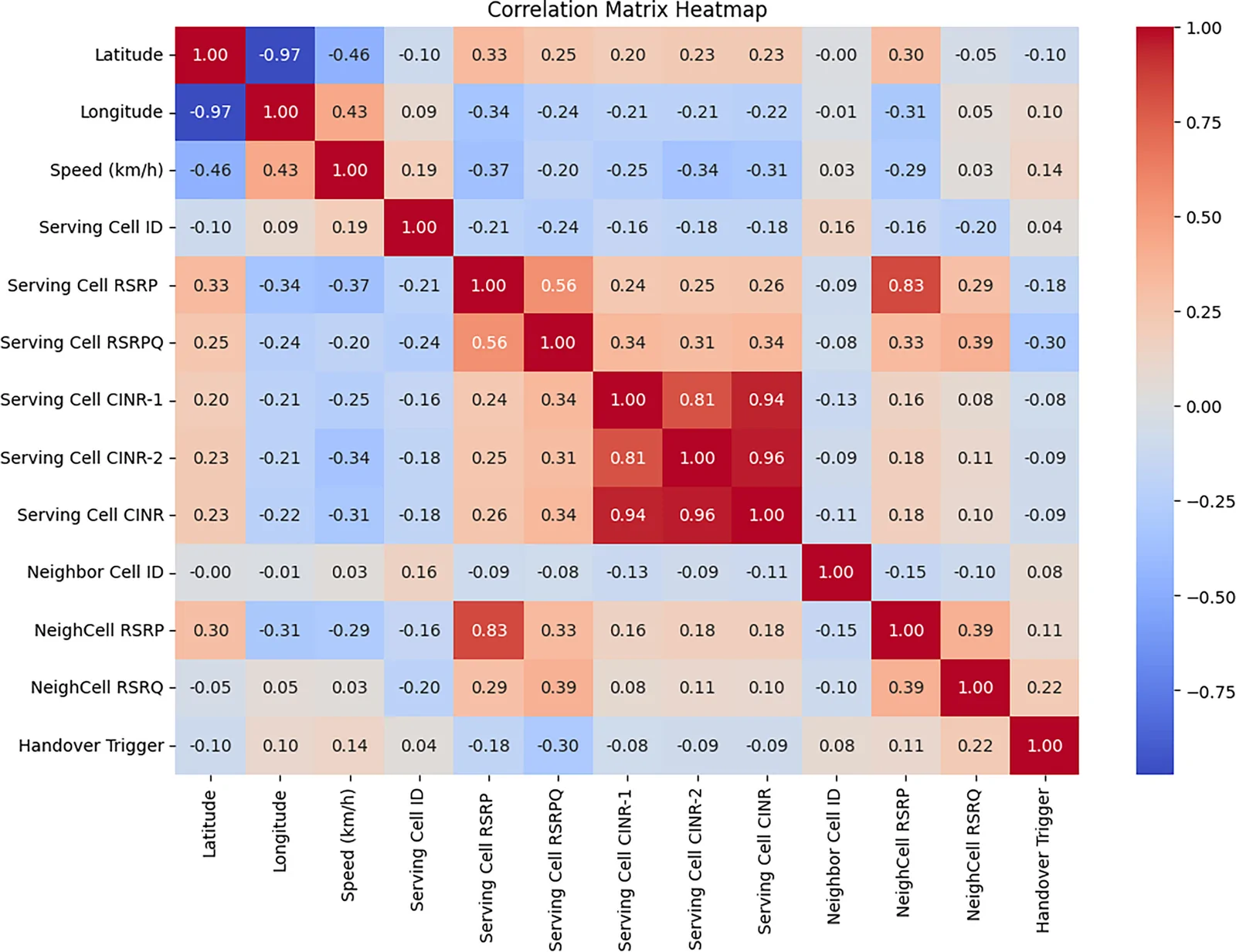
Correlation matrix illustrating the relationships among the network parameters and mobility variables in Dataset_3.

To understand the relationships between key signal parameters, correlation matrices were computed for each of the three datasets. These matrices help identify potential linear dependencies between features such as RSRP, RSRQ, CINR, and UE velocity, which are critical for modeling handover decisions. The correlation patterns are visualized in Fig. 1 for Dataset_1, Fig. 2 for Dataset_2, and Fig. 3 for Dataset_3. These visualizations provide a descriptive overview of relationships among key radio parameters and mobility indicators, supporting exploratory analysis of LTE signal behavior and handover conditions by highlighting strongly correlated or independent variables.

**Fig. 4:**
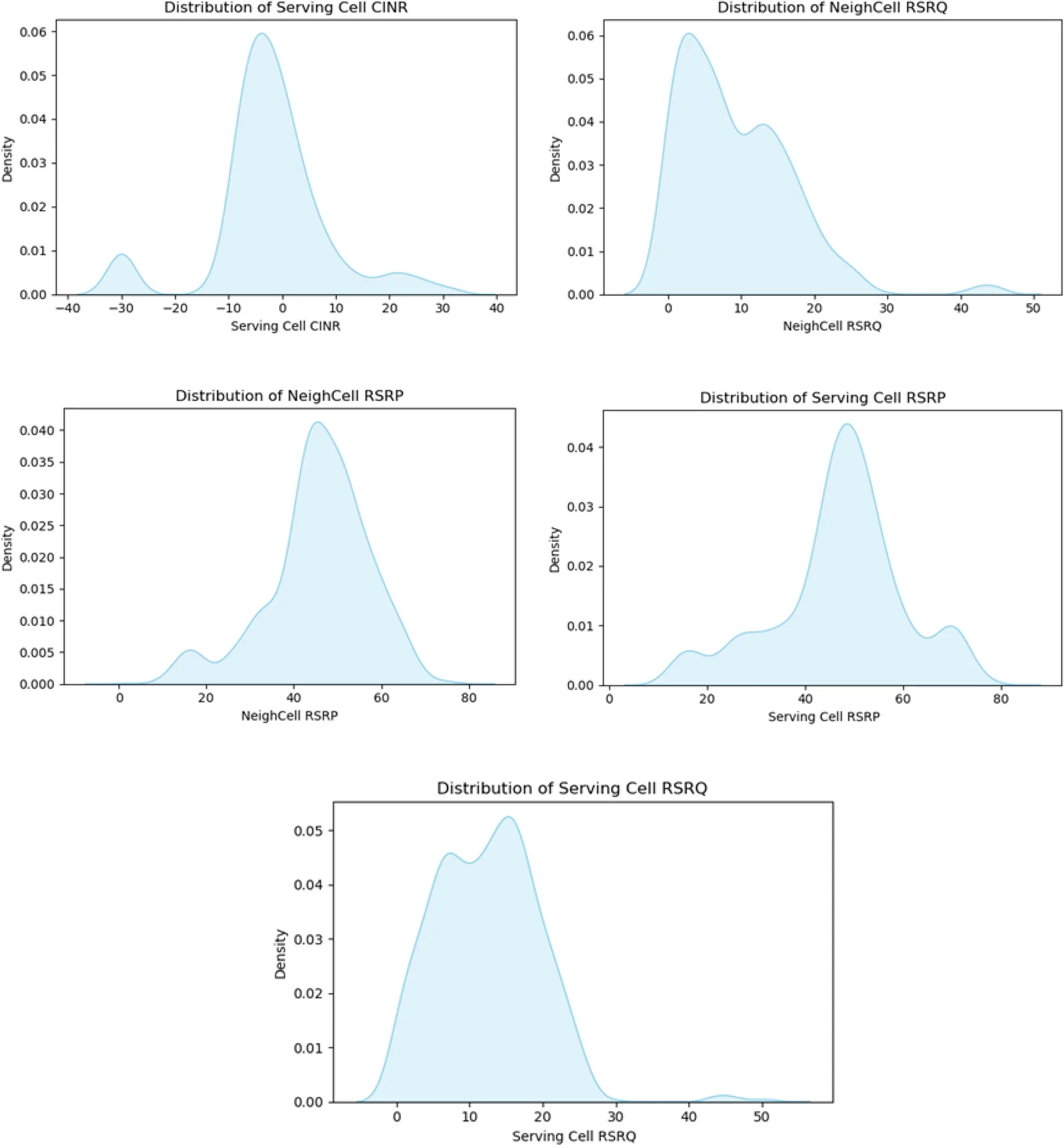
Distribution of network parameters of Dataset_1.

Additionally, the overall distribution of key network parameters, including RSRP, RSRQ and CINR, Dataset_1, is visualized in Fig. 4, providing insight into the signal environment and mobility conditions under which the data were collected.

### 3.3 LTE measurement report quantization ranges

In LTE networks, the Reference Signal Received Power (RSRP) and Reference Signal Received Quality (RSRQ) values reported by the User Equipment (UE) are quantized before being sent in measurement reports. These quantized values are represented by integer values according to 3GPP specifications [16].

The tables below illustrate the quantization mappings used for RSRP and RSRQ in the Measurement Reports included in our dataset. Understanding these mappings is essential for interpreting the raw Layer 3 signaling messages (e.g., MeasReport) extracted using XCAL-M software.

In LTE networks, signal quality metrics such as RSRP and RSRQ are quantized before being reported in Layer 3 measurement messages. The integer values assigned to these metrics follow specific mapping schemes defined by 3GPP standards. The quantization mapping for RSRP values is shown in Table 9, while the corresponding mapping for RSRQ values is presented in Table 10. These mappings are essential for interpreting the raw measurement reports contained in the dataset.

**Table 10:** RSRP quantization range.

RSRP-Range IE Value	RSRP Value
0	RSRP < −140 dBm
1	−140 dBm ≤ RSRP < −139 dBm
2	−139 dBm ≤ RSRP < −138 dBm
3	−138 dBm ≤ RSRP < −137 dBm
…	…
95	−46 dBm ≤ RSRP < −45 dBm
96	−45 dBm ≤ RSRP < −44 dBm
97	−44 dBm ≤ RSRP

The original radio measurements acquired by the XCAL-M drive-test software were recorded in their physical units, with RSRP expressed in dBm and RSRQ expressed in dB. During preprocessing, these measurements were converted to the standardized LTE Radio Resource Control (RRC) Information Element (IE) representations defined by the 3GPP specifications to ensure consistency with protocol-level measurement reporting and facilitate structured data analysis. Consequently, the released dataset stores the quantized IE values for RSRP and RSRQ rather than their original physical-unit measurements. The corresponding conversion between physical values and IE representations follows the mapping specified in the 3GPP LTE standards and is summarized in Table 10, Table 11.

**Table 11:** RSRQ quantization range.

RSRQ-Range IE Value	RSRQ Value
0	RSRQ < −19.5 dB
1	−19.5 dB ≤ RSRQ < −19 dB
2	−19 dB ≤ RSRQ < −18.5 dB
3	−18.5 dB ≤ RSRQ < −18 dB
…	…
32	−4 dB ≤ RSRQ < −3.5 dB
33	−3.5 dB ≤ RSRQ < −3 dB
34	−3 dB ≤ RSRQ

### 3.4 Drive-test DRM logs, measurement reports, and handover event statistics

In addition to the processed tabular datasets, the repository includes the original XCAL-M DRM log files, Layer-3 measurement report records, and handover event statistics extracted during the drive-test sessions. The inclusion of these low-level diagnostic records increases the technical depth and reproducibility of the dataset by preserving the original signaling information captured from the operational LTE network.

#### 3.4.1 DRM log files

The raw DRM files contain the exact diagnostic traces recorded by the XCAL-M drive-testing platform during data collection. These logs preserve the original network signaling and radio measurement information generated throughout the drive-test sessions, including radio resource control (RRC) procedures, serving and neighbor cell measurements, signaling exchanges, and handover-related events. Because the original DRM files are included without modification, researchers may independently reprocess the measurements, extract additional radio or protocol-level features, and apply alternative preprocessing pipelines beyond the processed datasets provided in this work.

The availability of raw diagnostic traces is particularly important for reproducibility and future extensibility of the dataset. Researchers working on LTE mobility analysis, handover optimization, radio environment characterization, or protocol-level investigations may directly retrieve low-level measurements from the DRM logs according to their own analytical requirements. Furthermore, these diagnostic records may serve as practical educational material for cellular communication coursework by allowing students and researchers to visualize real-world LTE signaling procedures and mobility behavior captured from an operational network environment.

#### 3.4.2 Handover event statistics

The dataset also includes structured handover event statistics exported from the XCAL-M event analysis module. These records contain timestamped information associated with mobility-related network events, including event type, carrier frequency, radio access/base type, handover attempt count, and handover success status. Such event-level information enables detailed examination of handover execution behavior during mobility scenarios.

The handover event statistics are useful for analyzing mobility-management performance, identifying successful and unsuccessful handover transitions, correlating radio conditions with handover outcomes, and examining temporal patterns of mobility events during the drive-test sessions.

#### 3.4.3 Layer-3 measurement reports

The repository additionally contains Layer-3 LTE signaling messages extracted from the diagnostic traces, including RRC messages such as paging records, connection reconfiguration procedures, and measurement reports. These signaling messages provide direct visibility into UE-network interactions occurring during LTE mobility operations.

Among these records, LTE *measurementReport* messages are particularly important because they contain serving-cell and neighbor-cell radio measurements reported by the UE to the network during mobility management procedures. These reports include parameters such as RSRP and RSRQ for both serving and neighboring cells together with physical cell identifiers and measurement identifiers associated with handover triggering conditions.

The inclusion of raw Layer-3 signaling messages makes the dataset technically distinctive compared with many publicly available LTE datasets that only provide aggregated KPIs or throughput measurements. By preserving the original protocol-level signaling records, the dataset enables more detailed analysis of LTE mobility procedures, handover triggering behavior, measurement reporting mechanisms, and radio resource management operations within real cellular network conditions.

The dataset is hosted on Mendeley Data as Version 2.0. Any future updates, corrections, or extensions will be released as subsequent repository versions to ensure version traceability and reproducibility. File versioning and integrity are managed through the Mendeley Data repository.

### 3.5 Data dictionary

To facilitate dataset interpretation and reuse, Table 12, Table 13 provide a consolidated data dictionary describing each variable included in the released datasets. The table summarizes the definition, data type, unit, value representation, and preprocessing history for each variable. Unless otherwise stated, missing values in the processed datasets were removed during preprocessing, while the raw diagnostic files preserve the original measurements.

**Table 12:** Data dictionary for the released datasets.

Variable	Description	Data Type	Unit / Representation	Notes
**Timestamp**	Time at which the radio measurement was recorded.	Timestamp	hh:mm:ss.sss	Used for synchronization and timestamp alignment during preprocessing.
**Longitude**	Geographic longitude of the UE location.	Float	Decimal degrees	Recorded from GPS. Available only for Datasets 2 and 3.
**Latitude**	Geographic latitude of the UE location.	Float	Decimal degrees	Recorded from GPS. Available only for Datasets 2 and 3.
**Speed (km/h)**	Instantaneous UE speed during the drive test.	Float	km/h	Recorded from GPS. Available only for Datasets 2 and 3.
**Serving Cell ID**	Physical Cell Identifier (PCI) of the serving LTE cell.	Integer	PCI	Directly extracted from XCAL-M measurement logs.
**Serving Cell RSRP**	Reference Signal Received Power of the serving cell.	Integer	3GPP RRC Information Element (IE) value	Originally recorded in dBm by XCAL-M and converted to standardized 3GPP IE values during preprocessing (Table 9, Table 10).
**Serving Cell RSRQ**	Reference Signal Received Quality of the serving cell.	Integer	3GPP RRC Information Element (IE) value	Originally recorded in dB by XCAL-M and converted to standardized 3GPP IE values during preprocessing (Table 9, Table 10).
**Serving Cell CINR-1**	Lower CINR measurement reported for the serving cell.	Float	dB	Directly extracted from XCAL-M diagnostic logs.
**Serving Cell CINR-2**	Upper CINR measurement reported for the serving cell.	Float	dB	Directly extracted from XCAL-M diagnostic logs.
**Serving Cell CINR**	Representative CINR of the serving cell computed as the arithmetic mean of CINR-1 and CINR-2.	Float	dB	Derived during preprocessing.
**Neighbour Cell ID**	Physical Cell Identifier (PCI) of the neighboring LTE cell reported in the Measurement Report.	Integer	PCI	Available only when neighboring-cell measurements were reported by the UE.
**Neighbour Cell RSRP**	Reference Signal Received Power of the neighboring cell.	Integer	3GPP RRC Information Element (IE) value	Converted from physical dBm values to standardized 3GPP IE values.
**Neighbour Cell RSRQ**	Reference Signal Received Quality of the neighboring cell.	Integer	3GPP RRC Information Element (IE) value	Converted from physical dB values to standardized 3GPP IE values.
**Handover Trigger**	Indicates whether a measurement corresponds to a handover-triggering instance.	Binary (0/1)	Boolean	Assigned during preprocessing using timestamp alignment and handover event matching.

**Table 13:** Data dictionary for event statistics.

Variable	Description	Data Type	Unit / Representation	Notes
**Time**	Timestamp of the recorded network event.	Timestamp	mm:ss.s	Exported directly from the XCAL-M Event Statistics module.
**Event**	Type of mobility or network event (e.g., Intra LTE-HO, Idle Mode Load Balancing).	String	Event label	Represents the detected LTE mobility event.
**Freq**	Frequency and radio system information associated with the event.	String	XCAL-M event format	Describes the source and target carrier/frequency information.
**Base**	Handover execution interface or network entity (e.g., eNB, X2).	String	LTE interface	Indicates the network entity responsible for the mobility procedure.
**Result**	Outcome or stage of the recorded event (e.g., Attempt or Success).	String	Event status	Used to identify handover progression.
**Attempt**	Number of handover attempts recorded for the event.	Integer	Count	Exported directly from the Event Statistics report.
**Success**	Number of successful handovers associated with the event.	Integer	Count	Exported directly from the Event Statistics report.
**Fail**	Number of failed handovers associated with the event.	Integer	Count	Zero when no failure occurred.
**Count**	Aggregate occurrence count reported by XCAL-M for the corresponding event.	Integer	Count	Included when available in the exported report.

## 4 Experimental Design, Materials and Methods

Long Term Evolution (LTE) is a fully packet-switched system comprising the Evolved Packet Core (EPC), eNodeB, and User Equipment (UE), designed under the System Architecture Evolution (SAE) to support seamless mobility and low-latency communication [17]. Together with the Evolved Universal Terrestrial Radio Access Network (EUTRAN), it forms the Evolved Packet System (EPS) [18].

To assess real-world mobility performance, drive tests are categorized as Single Site Verification (SSV), Multiple Site Verification (MSV), or Operator Benchmarking [9]. Our study conducted an MSV drive test as it was performed across a cluster of LTE cells along a 13 km urban route in Dhaka, characterized by frequent inter-cell handovers. The goal was to capture real LTE radio measurements and handover statistics along a representative urban route, reflecting real network behavior during the measurement sessions within dense urban deployments [19].

**Fig. 5:**
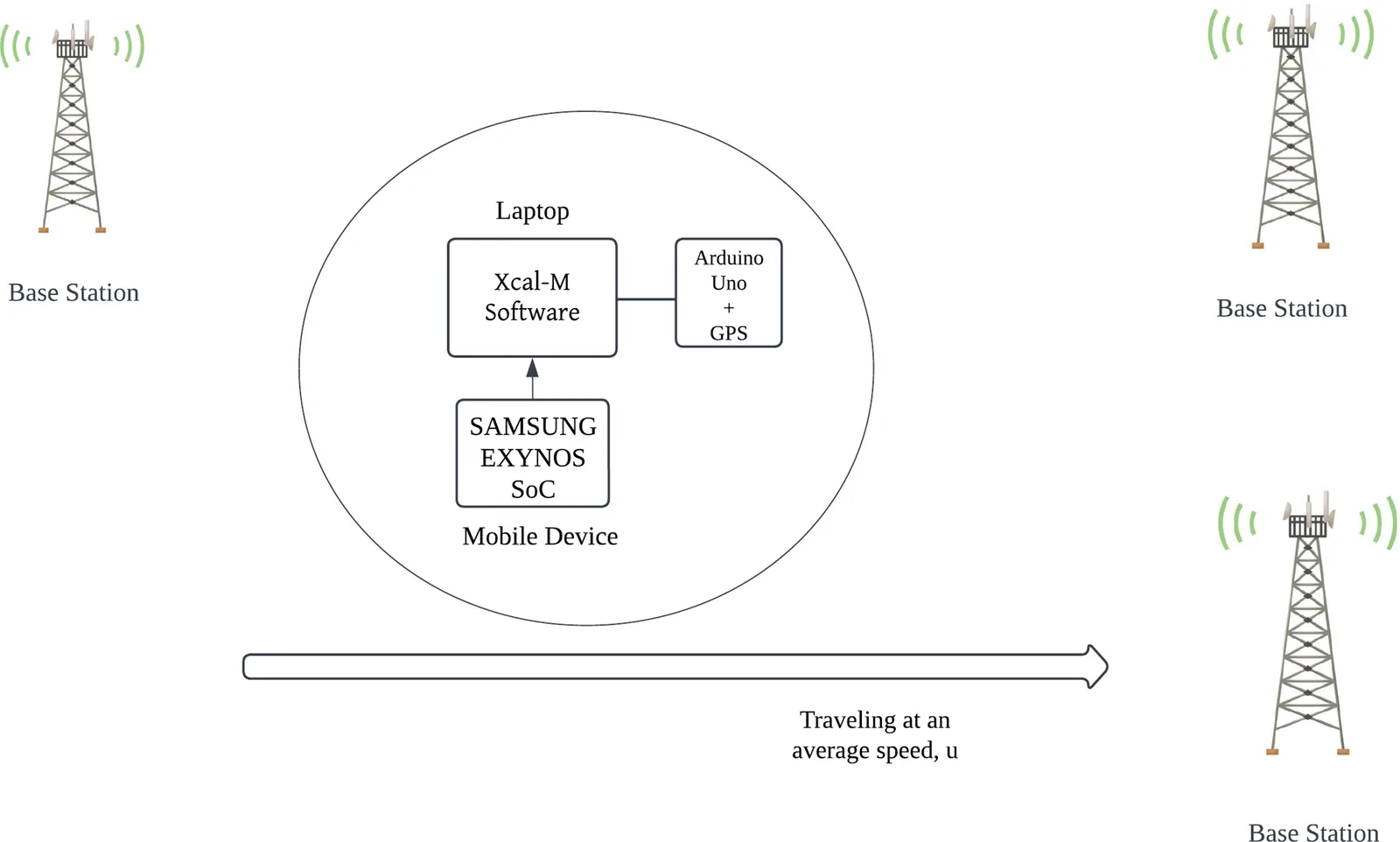
Schematic diagram of drive test.

This section encompasses details about the drive test location, time period, required equipment, software set up and data extraction methods. The overall drive test setup, including hardware and software components, is illustrated in Fig. 5.

### 4.1 Equipment used in the drive test

• XCAL-M software
• Samsung device with Exynos SoC chipset
• Laptop
• Android-based mobile phone
• Arduino Uno
• Neo-7 M GPS module
• Vehicle

### 4.2 Location of the drive test

To select an appropriate location for the drive test, we utilized the publicly available cell ID database from the OpenCellID website, which provides approximate geolocations of cellular base stations. This database includes information for multiple Radio Access Technologies (RATs), namely GSM, UMTS, and LTE. For the purpose of this study, which focuses on LTE handover behavior, we specifically filtered and targeted LTE eNodeBs [20]. We selected a region within Dhaka, Bangladesh characterized by a high density of LTE base stations to ensure frequent inter-cell handover events, which are critical for evaluating handover decision algorithms and mobility management performance.

The drive test route spanned approximately 13 km, beginning at Le Meridien Dhaka (Uttara) and concluding at the BRAC University campus. This route includes variations in user mobility patterns such as stationary periods, pedestrian speeds, and vehicular movement under mixed traffic conditions. This route was selected because it passes through a mix of dense commercial zones, educational centres, residential areas, and major intersections, making it highly representative of typical urban mobility patterns in Dhaka. Such contextual representativeness is crucial for meaningful mobility analysis and dataset generalizability in wireless studies [2]. The measurements were collected using the Grameenphone LTE network, one of the largest mobile network operators in Bangladesh.

**Fig. 6:**
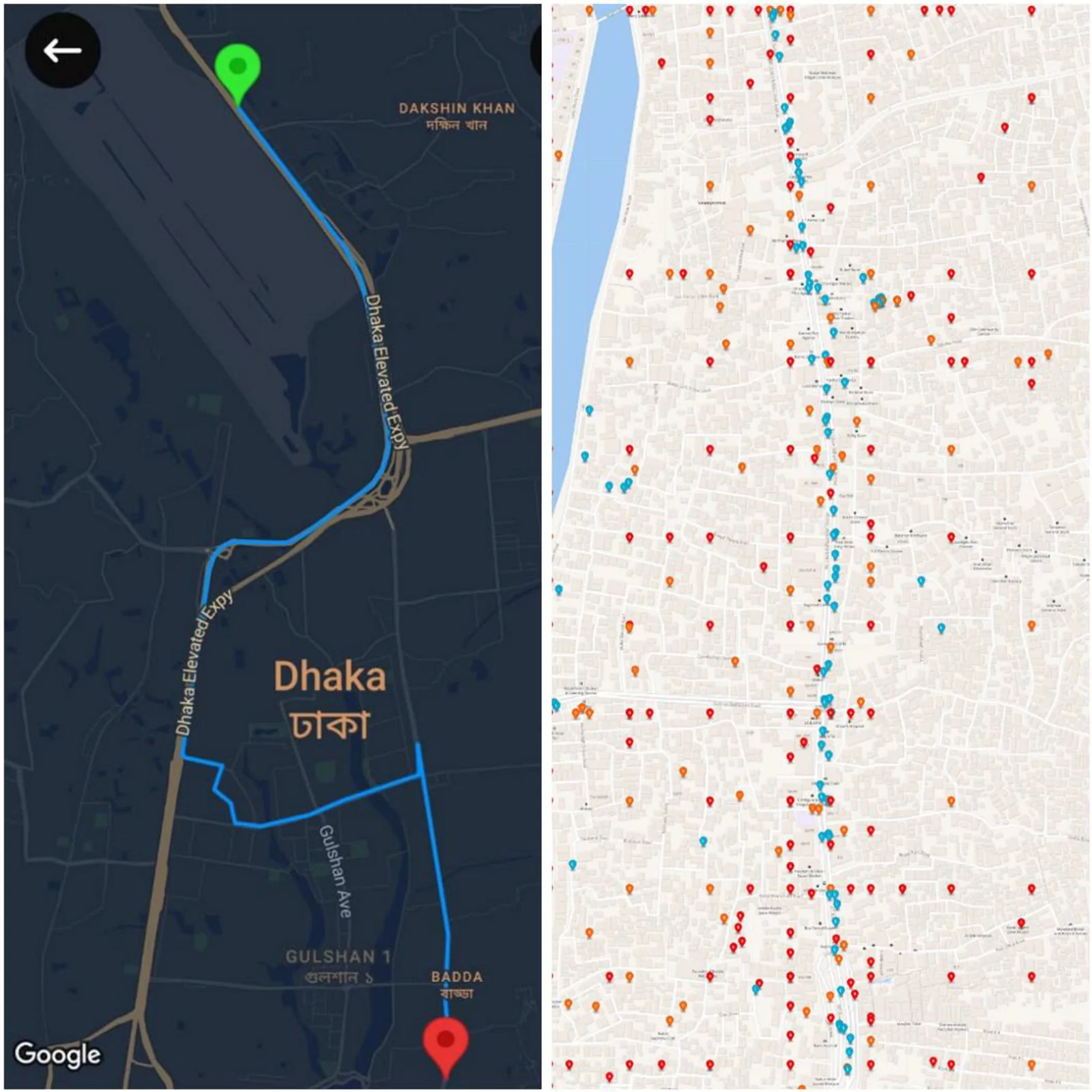
Drive test route on google maps (Left) and cell ID database from OpenCellID (Right).

It is important to note that, due to the specific selection of the test area the use of a single Mobile Network Operator (MNO), the results obtained from this drive test may not be fully representative of broader network conditions across the city. Additionally, the findings may not generalize to other network operators with differing infrastructure deployments, frequency bands, or mobility management strategies. The Cell ID mapping from OpenCellID and the corresponding drive test route on Google Maps are shown in Fig. 6.

### 4.3 Time period

The objective of the measurement campaign was to capture representative LTE radio conditions during typical urban traffic movement rather than to characterize long-term network behavior across all temporal scenarios. Consequently, the dataset represents a snapshot of network conditions during specific drive-test sessions rather than a comprehensive temporal survey of the cellular network.

The drive test was conducted over three separate days: October 15, November 6, and November 16, with measurements recorded during the evening hours between 16:00 and 22:00 (local time). On each day, the test route—spanning from Le Meridien Dhaka (Uttara) to BRAC University and back—was traversed in two complete rounds, with a 30-minute interval between each outbound and return trip. The time window was deliberately chosen to coincide with evening peak hours, a period characterized by high user density and network load, which can significantly influence radio resource management and handover dynamics. The selected route lies in one of the busiest corridors of Dhaka city, commonly subject to vehicular congestion and traffic delays. Such conditions introduced non-uniform mobility patterns (e.g., stop-and-go movement), which in turn affect radio measurements, particularly those related to handover triggering, RSRP fluctuation, and SINR degradation due to prolonged exposure to edge-of-cell conditions.

Traffic-induced delays allowed for denser spatial sampling of radio conditions, especially near cell edges. The short, repeatedly traversed route led to multiple encounters with the same LTE cell IDs under varying conditions, enhancing the dataset’s temporal diversity. This redundancy improves the robustness and generalizability of machine learning models for handover prediction.

The network infrastructure in the area remained operationally static during the measurement period; that is, no observable commissioning or decommissioning of LTE eNodeBs took place. Therefore, temporal variations in collected Key Performance Indicators (KPIs) [21] are attributed primarily to changes in user mobility and traffic conditions, rather than alterations in network topology or configuration. The average ambient temperature and relative humidity during the test periods were approximately 29 °C and 72%, respectively—values typical of Dhaka's tropical climate in the post-monsoon season.

### 4.4 Software set up

For the execution of the drive test, we employed XCAL-M (version O3.5.2.64 N), a professional-grade drive test software developed by Accuver, installed on a laptop and interfaced with a Samsung Galaxy S10 device powered by the Exynos chipset. This setup enabled the collection of detailed Layer 1 to Layer 3 signaling information, radio frequency (RF) metrics, and protocol-level data essential for in-depth analysis of mobility and handover performance in LTE networks. The mobile device was connected via diagnostic mode to ensure direct access to chipset-level KPIs.

In addition, we integrated an Arduino Uno microcontroller with a Neo-7 M GPS module to continuously log the user equipment’s geolocation (Latitude and Longitude) and UE velocity. This auxiliary setup ensured accurate tracking of mobility patterns, enabling correlation between user movement and observed network behavior.

The GPS device recorded timestamped latitude, longitude, and velocity measurements at regular intervals during the drive-test sessions. Because the GPS receiver was positioned inside the vehicle rather than roof-mounted, the measurements may be affected by signal attenuation caused by vehicle structure and urban canyon effects. As a result, minor positional inaccuracies may occur in dense urban environments. For this reason, the geographic coordinates should be interpreted as approximate indicators rather than high-precision positioning data. The primary objective of the dataset remains the documentation of LTE radio measurements and handover behavior, while the GPS-derived variables provide contextual information about user mobility during the measurement sessions. For applications requiring higher geospatial accuracy, future data collection is recommended to employ a roof-mounted external GNSS antenna or professional survey-grade positioning system to minimise signal attenuation and multipath effects.Compared to Android-based applications such as G-NetTrack Pro, which typically record measurements at one-second intervals due to OS-level constraints, XCAL-M offers millisecond-level granularity, making it substantially more suitable for fine-grained temporal and spatial analysis of handover events and transient radio conditions [22].

Prior to each drive-test session, the laptop, Samsung device and GPS logger were synchronized to the same system time. GPS acquisition was verified after obtaining a stable satellite fix before data collection commenced. No additional calibration of the XCAL-M radio measurements was required because the software directly records chipset-level diagnostic measurements from the commercial LTE user equipment.

The Samsung S10 device, serving as the user equipment (UE), was placed inside a private test vehicle, and the entire system was configured for real-time data acquisition as the vehicle traversed the defined route. Throughout the drive, XCAL-M continuously captured network performance metrics and handover signaling messages, synchronized with precise GPS coordinates and time stamps.

### 4.5 Data extraction from XCAL-M

**Fig. 7:**
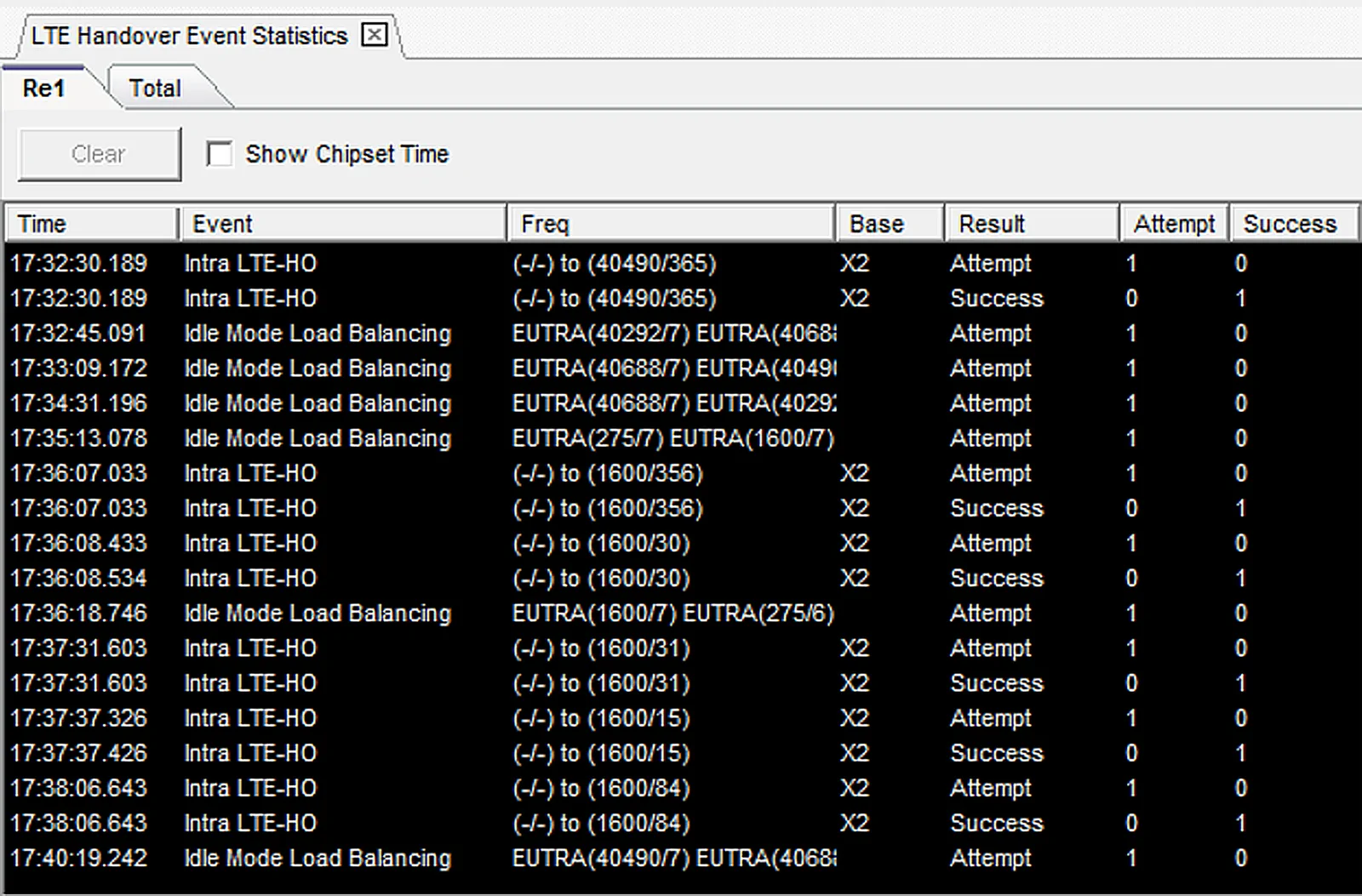
LTE handover event statistics from X-CAL M software.

Following the completion of the drive test, network performance data was extracted using the built-in post-processing capabilities of XCAL-M. To analyze mobility and handover-related events, we navigated to the “Layer3 KPI” module, selected “LTE”, and then accessed the “LTE Handover Event Statistics” window. This interface provided detailed handover metrics, including the number of handover attempts, successful handovers, and corresponding event types along with precise timestamps, enabling temporal correlation with user movement and signal conditions. The statistics are visualized in Fig. 7.

**Fig. 8:**
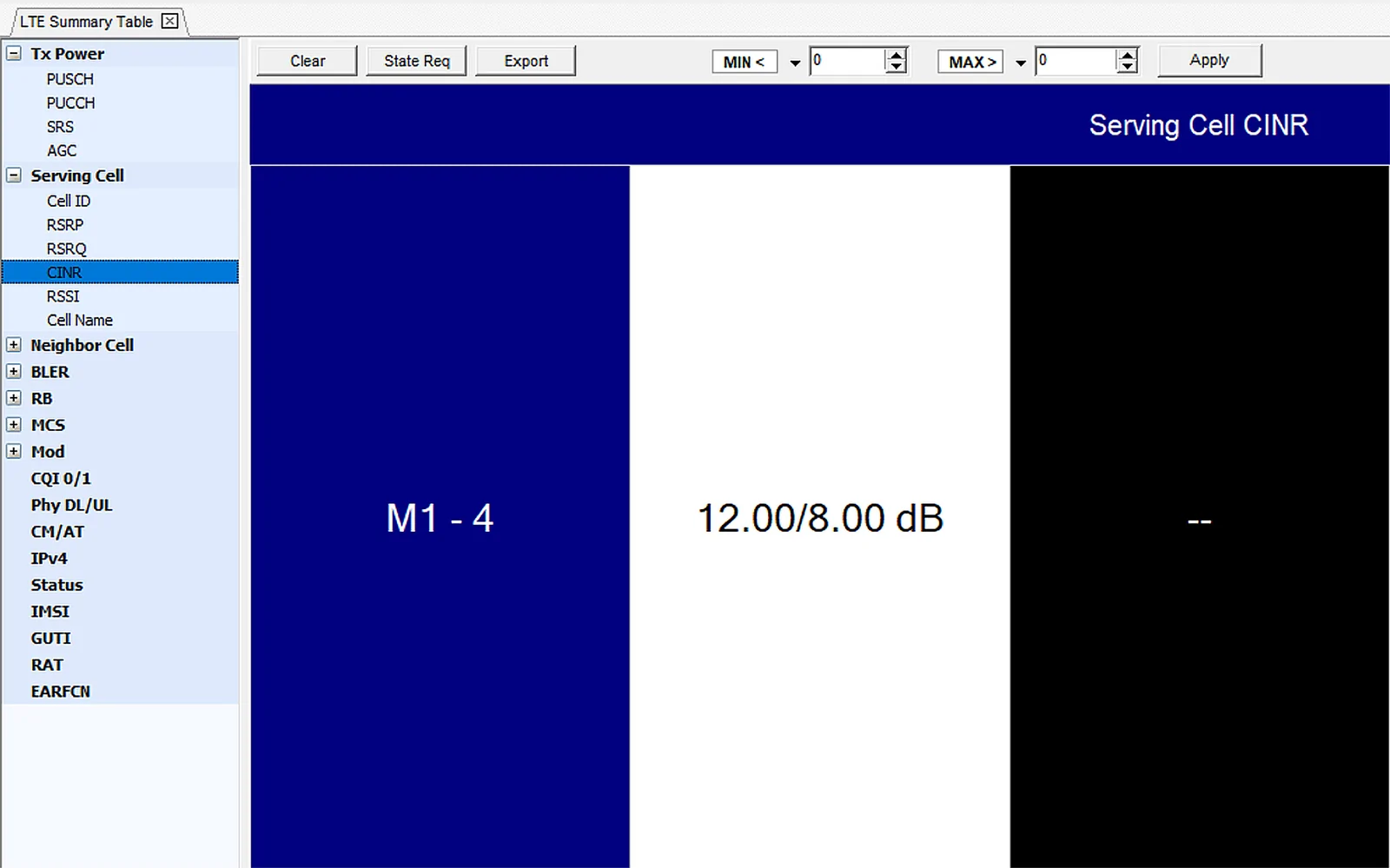
LTE summary table from X-CAL M software.

For extracting information on serving cell parameters, we utilized the “Call Statistics” option, selected the “LTE Summary Table”, and further filtered the data by choosing the “Serving Cell” view. This yielded essential serving cell identifiers, including the Physical Cell ID (PCI) and Carrier-to-Interference-plus-Noise Ratio (CINR). LTE Summary Table is visualized in Fig. 8.

**Fig. 9:**
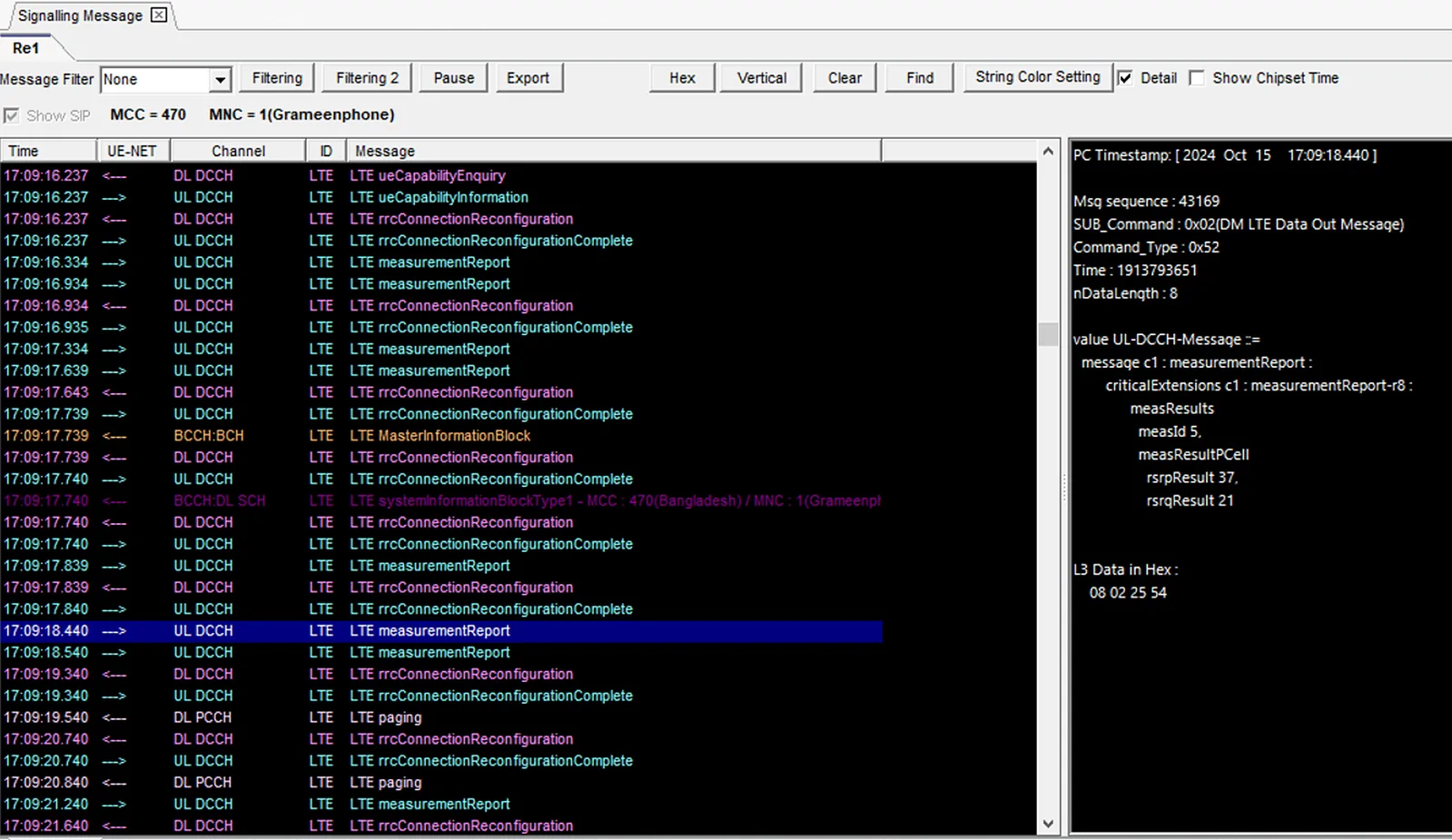
LTE signaling message from X-CAL M software.

To obtain detailed radio measurement data, we accessed the “Message” tab, selected “Signaling Message”, and filtered for “Measurement Report” messages. These messages included Serving Cell RSRP, RSRQ, and corresponding Neighbor Cell RSRP, RSRQ, along with Neighbor Cell IDs, all timestamped for precise temporal alignment. Measurement reports play a crucial role in LTE networks, as they are fundamental to the handover decision-making process, enabling the eNodeB to evaluate surrounding cell conditions and initiate mobility procedures based on preconfigured event thresholds. A visualization of LTE Signaling Message is given in Fig. 9.

In LTE, Reference Signal Received Power (RSRP) and Reference Signal Received Quality (RSRQ) are critical physical layer metrics reported by the User Equipment (UE) in Layer 3 Measurement Reports, which are governed by the MeasConfig Information Element (IE). The S-Measure field within MeasConfig defines the RSRP threshold below which the UE initiates neighbor cell measurements. If S-Measure = 0, the UE is configured to perform neighbor measurements continuously, regardless of the serving cell’s RSRP level.

RSRP and RSRQ thresholds are quantized using RSRP-Range and RSRQ-Range IEs, respectively. The RSRP-Range IE supports integer values from 0 to 97, each corresponding to a 1 dB interval from below −140 dBm up to and beyond −44 dBm. The RSRQ-Range IE spans 0 to 34, mapping to standard RSRQ intervals. These quantized values appear in the Measurement Report under the signaling messages tab in XCAL-M software.

**End-to-End Data Processing Workflow**

1. Configure the Samsung Galaxy S10 in LTE diagnostic mode and connect it to XCAL-M.
2. Initialize the Neo-7 M GPS module and verify stable GPS reception.
3. Conduct the drive test while XCAL-M continuously records DRM diagnostic logs and Layer-3 signaling messages.
4. Export Measurement Reports, LTE Summary Tables, and Event Statistics from XCAL-M.
5. Extract raw CSV radio measurements from the exported logs.
6. Synchronize GPS and radio measurements using timestamps.
7. Apply cell-aware interpolation and generate TTT-based handover labels.
8. Release both the parent datasets and processed datasets in the public repository.

### 4.6 Data preprocessing

The objective of applying ML to the drive test dataset is to model and predict handover decisions based on real-world LTE mobility patterns. In this context, the handover serves as the target variable, while the number of handovers is used as the primary performance metric, with a focus on minimizing unnecessary handovers and ping-pong events [23] through reinforcement learning-based optimization to enhance network stability and efficiency. To prepare the dataset for ML models, a series of preprocessing steps were applied: data segments with hardware anomalies were excluded, and all non-LTE measurements were filtered out. Furthermore, the concept of TTT-a standardized delay (set as 320 ms in our study) before a handover is executed-was incorporated. While raw handover labels were derived directly from Cell ID transitions in the Event Statistics log, the refined dataset assigns a handover label to those datapoints that contribute to a handover decision within the TTT window, thereby capturing the pre-handover context necessary for accurate model training. An algorithm is developed below for TTT-Based Handover Label Generation: Algorithm 1

**Algorithm 1:** TTT-based handover label generation.

Require: Processed LTE measurement dataset D Event Statistics log E Time-to-Trigger (TTT) = 320 ms Ensure: TTT-aware handover labels 1: Initialize Handover Trigger = 0 for all records in D. 2: For each handover event e ∈ E do 3: Extract the handover occurrence timestamp t_HO_. 4: Define the TTT observation window W = [t_HO_ − TTT, t_HO_]. 5: Identify the measurement records within W that satisfy the handover decision criteria based on the recorded serving-cell and neighbour-cell measurements. 6: Assign Handover Trigger = 1 to the identified records. 7: End For 8: Return the updated dataset D.

In this study, a TTT value of 320 ms was adopted because it is one of the standardized LTE TTT values defined by 3GPP specifications and represents a commonly used compromise between handover responsiveness and signalling stability. Very short TTT values may increase unnecessary or ping-pong handovers, whereas excessively long values may delay legitimate handovers and increase the risk of radio link degradation. The selected value was therefore used to construct a representative pre-handover observation window for the processed dataset. Although LTE supports multiple standardized TTT values (0–5120 ms), investigating alternative configurations was beyond the scope of this dataset paper.

The larger reduction from the Parent Dataset in the processed size of Dataset 2 (∼61%) is primarily attributable to the preprocessing strategy rather than data acquisition quality. LTE radio measurements were extracted from Layer-3 Measurement Report messages, in which neighboring-cell measurements are reported by the UE only when they satisfy network-configured measurement criteria, such as becoming sufficiently relevant for mobility evaluation or potential handover decisions. Consequently, many Measurement Reports in Dataset 2 contained only serving-cell measurements without corresponding neighboring-cell RSRP and/or RSRQ values. Because neighboring-cell information is essential for constructing handover-oriented feature vectors, records exhibiting extended consecutive intervals without neighbor-cell measurements were excluded during preprocessing. Therefore, the greater reduction observed in Dataset 2 reflects the characteristics of the Measurement Report signaling and the task-specific preprocessing pipeline, rather than missing raw measurement data.

In drive-test diagnostic logs, serving-cell and neighbour-cell measurement reports may appear asynchronously because they originate from different Layer-3 signalling messages within the LTE protocol stack. Consequently, certain signal parameters (e.g., RSRP or RSRQ) may be absent from individual timestamp entries. To generate the processed datasets, interpolation was performed using a cell-aware timestamp-aligned interpolation procedure rather than a conventional linear or spline interpolation technique. Specifically, measurements were first grouped according to their serving or neighbour cell identifiers (PCI/Cell ID). For each cell, temporally consecutive records with small timestamp gaps were examined, and missing signal measurements were populated using the most recent valid measurement belonging to the same cell when continuity of the cell association was preserved. Interpolation was applied only to short consecutive gaps and was not performed across cell transitions or prolonged missing intervals. This procedure preserves the temporal consistency of LTE measurement reports while avoiding interpolation across different radio environments. Approximately 11%, 15%, and 9% of the signal measurements in Datasets 1, 2, and 3, respectively, were interpolated, while the remaining measurements were obtained directly from the recorded LTE measurement reports. Because interpolation can introduce bias in small datasets, the processed dataset should be interpreted as an example representation of the measurement logs rather than a ground-truth reconstruction of radio conditions.

GPS records and radio measurements were acquired independently during the drive tests and subsequently synchronized manually through timestamp alignment. Matching was performed using the recorded timestamps to associate radio measurements with the corresponding mobility information before preprocessing. No statistical outlier detection or removal was performed because the objective was to preserve the original field measurements acquired during the drive tests. Duplicate measurement records were not observed during dataset preparation; therefore, no duplicate removal procedure was required. Apart from the conversion of RSRP and RSRQ to standardized 3GPP Information Element (IE) values and the calculation of the representative CINR value, no additional feature engineering or normalization was applied.

It is important to emphasize that the preprocessing workflow is illustrative rather than mandatory. Researchers requiring strict measurement fidelity may instead analyze the raw drive-test logs provided with the dataset or may alternatively process the raw logs using their own methodologies depending on the intended analysis.

### 4.7 Measurement metadata

Table 14 summarizes the key metadata associated with the data acquisition process to facilitate dataset interpretation and reuse.

**Table 14:** Measurement metadata.

Metadata	Description
Drive-test software	XCAL-M version O3.5.2.64N
Timestamp resolution	1 ms (millisecond precision)
Measurement logging	Continuous logging throughout each drive-test session
Sampling mechanism	Event-driven continuous logging based on LTE signaling and XCAL-M measurement reporting
GPS receiver	u-blox Neo-7 M GPS module
Synchronization method	GPS measurements were recorded separately using the Neo-7 M module and synchronized with the XCAL-M radio measurements through timestamp alignment during preprocessing.
Coordinate Reference System (CRS)	World Geodetic System 1984 (WGS 84, EPSG:4326) using decimal latitude and longitude coordinates

The timestamp resolution and continuous measurement logging enable fine-grained temporal analysis of LTE radio measurements. GPS and radio measurement records were synchronized during preprocessing using their timestamps, as described in Section 4.6.

## Limitations

Several limitations should be considered when using this dataset.

First, the dataset represents a relatively small number of observations compared with large-scale operator datasets. As a result, it is better suited for benchmarking studies, reinforcement-learning-based mobility experimentation, handover behavior analysis, and educational or reproducibility-focused cellular network research rather than training complex deep learning models.

Second, the dataset was collected from a single mobile network operator within one urban environment in Dhaka, Bangladesh. Consequently, the radio conditions and handover behavior observed in the dataset may not generalize to other networks or geographic regions.

Third, the dataset covers three drive-test sessions conducted during evening hours. Therefore, it does not represent the full range of temporal traffic conditions that may occur in the network.

Despite these limitations, the dataset provides transparent access to real LTE drive-test measurements together with raw diagnostic logs that enable independent analysis and reproducibility.

## Ethics Statement

The authors confirm that they have read and followed the ethical requirements for publication in Data in Brief. The current work does not involve human subjects, animal experiments, or any data collected from social media platforms.

## CRediT Author Statement

**M.M. Sadman Shafi:** Conceptualization, Methodology, Software, Formal analysis, Resources, Data Curation, Writing - Original Draft, Writing - Review & Editing. **K M Istiaque:** Conceptualization, Validation, Resources, Visualization, Data Curation, Writing - Review & Editing. **Shafayet Sadik Sowad:** Formal analysis, Software, Validation, Resources, Data Curation. **Tasnia Siddiqua Ahona:** Data Curation, Writing - Review & Editing. **Mubtasim Labib:** Data Curation. **Yasin Hasan Saad:** Data Curation. **Md Nazakat Eram:** Data Curation. **Mohammad T. Kawser:** Supervision, Project administration.

## Declaration of Generative AI and AI-assisted Technologies in the Writing Process

During the preparation of this work the authors used ChatGPT to assist in improving the readability and language of the manuscript. After using this tool, the authors carefully reviewed and edited the content as needed and take full responsibility for the content of the published article.

## Declaration of Competing Interest

The authors declare that they have no known competing financial interests or personal relationships that could have appeared to influence the work reported in this paper.

## Data Availability

Mendeley DataDrive-Test-Based LTE Handover Dataset for Cellular Mobility Studies in Urban Bangladesh (Original data)
